# Blood Clotting and the Pathogenesis of Types I and II Hereditary Angioedema

**DOI:** 10.1007/s12016-021-08837-6

**Published:** 2021-05-06

**Authors:** Steven de Maat, Kusumam Joseph, Coen Maas, Allen P. Kaplan

**Affiliations:** 1 CDL Research, University Medical Centre Utrecht, Utrecht University, Utrecht, the Netherlands; 2 BioCryst Pharmaceuticals, Inc., Durham, NC USA; 3 Department of Medicine, Medical University of South Carolina, Charleston, SC USA

**Keywords:** Angioedema, Coagulation, Bradykinin, Contact activation

## Abstract

The plasma contact system is the initiator of the intrinsic pathway of coagulation and the main producer of the inflammatory peptide bradykinin. When plasma is exposed to a negatively charged surface the two enzymes factor XII (FXII) and plasma prekallikrein (PK) bind to the surface alongside the co-factor high molecular weight kininogen (HK), where PK is non-covalently bound to. Here, FXII and PK undergo a reciprocal activation feedback loop that leads to full contact system activity in a matter of seconds. Although naturally occurring negatively charged surfaces have shown to be involved in the role of the contact system in thrombosis, such surfaces are elusive in the pathogenesis of bradykinin-driven hereditary angioedema (HAE). In this review, we will explore the molecular mechanisms behind contact system activation, their assembly on the endothelial surface, and their role in the HAE pathophysiology.

## Introduction

The plasma contact system is an enzymatic system involved in the formation of the inflammatory peptide bradykinin (BK) and blood coagulation in the vascular system. At its core, the system is composed of the two enzymes factor XII (FXII) and plasma prekallikrein (PK). Together with the non-enzymatic co-factor high molecular weight kininogen (HK), this system is often referred to as the kallikrein-kinin system (KKS).

### Major Constituents of the Plasma Contact System

#### Factor XII

Factor XII (FXII) is an 80-kDa serine protease which circulates at approximately 30 μg/mL (37 nM). It consists of six domains: Fibronectin type II, EGF-like 1, Fibronectin type I, EGF-like 2, Kringle, and the catalytic peptidase domain, which are kept in conformation via 20 disulfide bonds. FXII is heavily glycosylated with two N-linked and seven O-linked glycosylation sites; the latter affected in mutant FXII in many patients with nC1-INH-HAE, i.e., HAE with normal C1 esterase inhibitor (C1-INH). Between the Kringle and the protease domain, a region rich in proline residues is found. This proline-rich region is unique to FXII and contains all but one of the O-linked glycosylation sites. Changes in its glycosylation increase the sensitivity of FXII for activation by the negatively charged polymer dextran sulfate [1]. After production by the liver, FXII is secreted into the blood plasma. There is growing evidence for a separate pool of leukocyte-expressed FXII that contributes to wound healing and angiogenesis [2], but its contribution to HAE remains to be identified.

#### Plasma Prekallikrein/Kallikrein

Plasma prekallikrein (PK) is an 88-kDa serine protease and consists of four apple domains together with a catalytic peptidase domain, containing 18 disulfide bonds and only 5 N-linked glycosylation sites. Those apple domains are found in only one other plasma protein, namely, coagulation Factor XI (FXI). Plasma prekallikrein is predominantly produced and secreted by the liver (50 μg/mL in plasma; 581 nM), but PK production has also been found, to a minor extent, in cells of the epithelial kidneys, adrenal gland, and placenta [3–5]. While PK is produced as a monomeric enzyme, in plasma 75–80% is found to be in complex to its non-enzymatic co-factor high molecular weight kininogen (HK) [6, 7]. Although PK shares a high homology with FXI, FXI circulates primarily as a disulfide-linked homodimer, while PK is a monomer.

#### Kininogens

The transcription of the *KNG1* gene leads to various forms of kininogen as a result of alternative splicing at exon 10 [8]. The largest (120 kDa) splice variant is annotated as HK and is predominantly produced and secreted by the liver (70 μg/mL in plasma; 636 nM). It consists of three cystatin kininogen-type domains and three histidine-rich repeats. The first two “cystatin” regions actually can function as a protease inhibitor, for example, inhibition of the cysteine-protease, papain, or some cathepsins. Furthermore, HK contains four N-linked and eight O-linked glycosylation sites. In plasma, HK is found to be in complex with 75–80% PK [6, 7] and 95% of FXI [9, 10]. However, it does not circulate in complex with FXII. While HK lacks an enzymatic domain, it is essential to bring PK and FXI to activating surfaces. Furthermore, it is the parent molecule from which the BK peptide is liberated. A smaller variant (64 kDa), annotated as low molecular weight kininogen (LK), lacks the three histidine-rich repeats and all but one of the O-linked glycosylation sites. Moreover, LK does not bind to negatively charged surfaces, PK or FXI as it misses the essential light chain for these interactions [10, 11]. In plasma, cleavage of HK by active plasma kallikrein (PKa) leads to the release of BK (nine amino acids; RPPGFSPFR) [12]. In tissues, cleavage LK by tissue kallikreins leads to the release of kallidin (lysyl-BK; contains one additional N-terminal lysine; 10 amino acids; KRPPGFSPFR), which can be cleaved into BK via aminopeptidase P. Nonetheless, LK is considered to be irrelevant for the formation of BK by the plasma contact system. Plasma concentrations of FXII, PK, and HK have shown to be partially dependent on estrogen-levels [13].

#### Surfaces

When plasma contacts an activating surface, the contact system will assemble on it. Where FXII is able to directly bind to these surfaces, PK requires HK for binding. The binding of FXII to anionic surfaces is a key to initiating the contact activation. The predominant binding site for kaolin (a diagnostic reagent that triggers FXII activation in the aPTT in-vitro assay) and polyphosphate nanoparticles (complexed to calcium) is the EGF-1 domain [14].

Surface binding stimulates FXII auto-activation; a process in which low-level proteolytic activity (1:56,000 the activity of activated FXII (FXIIa)) is amplified through surface concentration, conformational changes [15] and enzymatic crosstalk in which molecules of FXIIa cleave more FXII zymogen to activate it. Although activating surfaces have been pinpointed in the context of thrombosis and allergic reactions (reviewed in [16, 17]), these are still elusive in the pathogenesis of HAE.

**Fig. 1:**
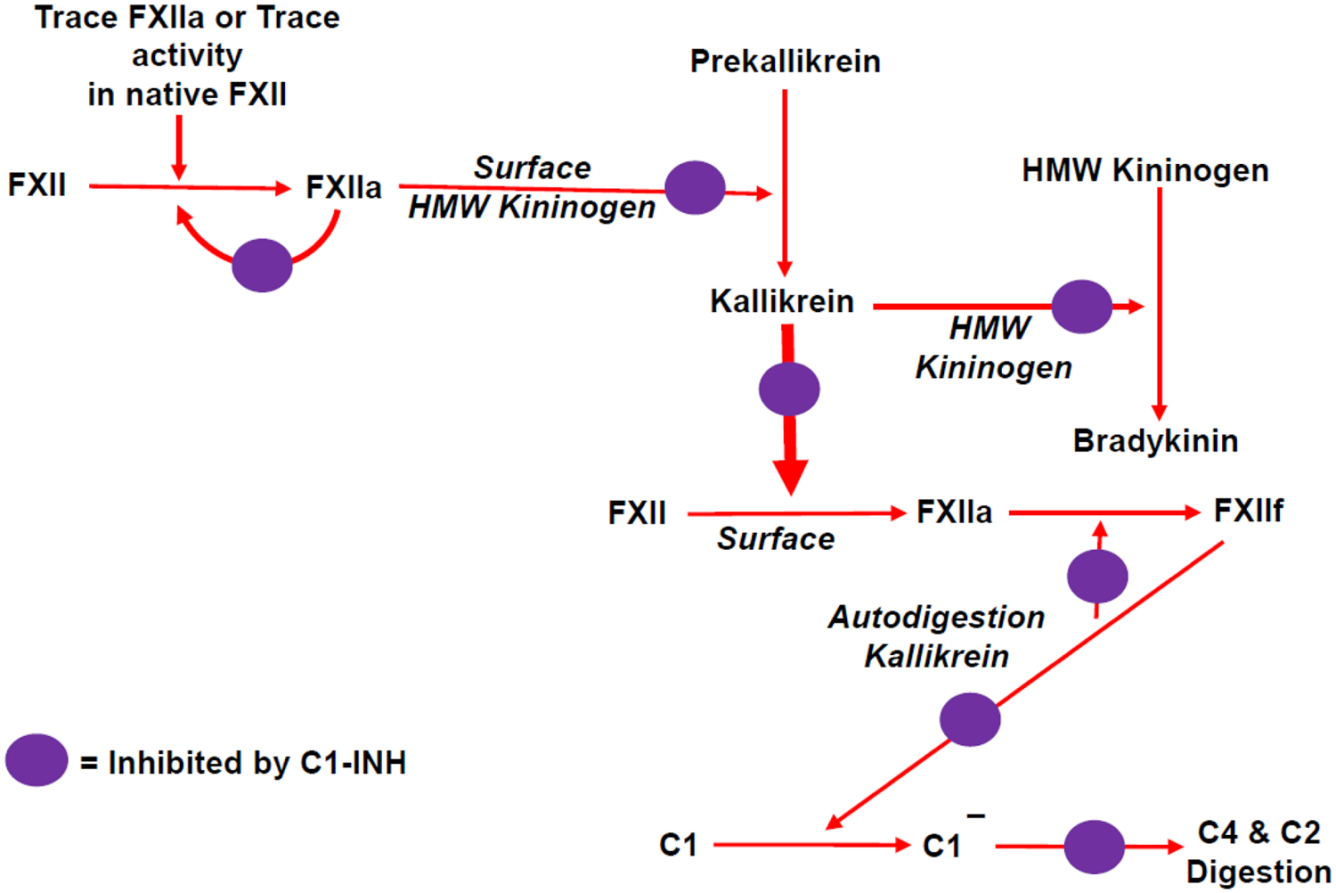
The mechanism of bradykinin formation in types I and II HAE and sites of inhibition by C1-INH. The steps include autoactivation of FXII, conversion of prekallikrein (PK) to kallikrein (PKa), the PKa “feedback” for rapid activation of FXII, and cleavage of HK to liberate BK. Further cleavage of FXIIa by PKa yields FXIIf (βFXIIa) which loses its ability to activate FXII but gains the ability to activate C1r and to a lesser degree C1s, thereby further depleting C4 and cleaving (activating) C2 during attacks of swelling

After auto-activation, FXIIa will activate PK, which reciprocally activates more FXII (Fig. 1). Activated PK will also cleave HK, thereby liberating BK from HK. This aggressive reciprocal activation feedback loop between FXII(a) and PK(a) allows for complete activation of the contact system in a matter of minutes [18]. Activated FXII exists in at least two distinct forms: αFXIIa (80 kDa) and βFXIIa (28 kDa; first described as FXIIf) [19, 20]. αFXIIa retains the capacity to bind to activating surfaces and activate both PK and FXI. In contrast, βFXIIa loses its capacity for binding, but readily activates PK in fluid-phase, but not FXI [21]. The trace coagulant activity ascribed to βFXIIa, i.e., FXIIf [19], estimated as 2–4% of αFXIIa [21], is due to trace activation of FXI or indirect Factor IX (FIX) activation by PKa. βFXIIa is a downstream cleavage product (because of PKa-mediated cleavage behind arginine 334) and comprises the catalytic domain together with a disulfide-linked peptide (335–343 or 335–353) which are formed sequentially, but rapidly, so a mixture is typically present [22]. This peptide is essential to the catalytic function of FXIIa, as removal of this remnant of the original heavy chain pushes the catalytic domain into a zymogen-like state [23].

A critical step in FXII activation is the displacement of the Fibronectin type II domain. In solution, this domain protects a cleavage site that is critical to FXII activation. Surface binding exposes this cleavage site [14]. Recently, a family was identified in which a mutation caused constitutive exposure of the activating cleavage site, resulting is a clinical picture of cold-induced urticarial syndrome [24].

During the 1970s and 1980s, the outlines of the BK-forming cascade were developed. The activated forms of FXII were described [19, 21], PK was shown to exist [19], then PK was purified and its mechanism of activation delineated [25]. Cleavage of kininogen by PKa was known in the 1960s but there was a debate (particularly regarding human plasma) as to how many kininogens were present although most authors favored two forms differing by size [26]. Ultimately two were distinguished structurally and functionally, facilitated by the discovery of plasma deficient in total kininogen [27] or just the high molecular weight form [28]. C1-esterase inhibitor (C1-INH) was found to inhibit purified PKa [29], and both forms of FXIIa [30, 31] as well as FXIa [30]. Although C1-INH is the dominant physiological inhibitor of these enzymes, it is not a very strong inhibitor (at a kinetic level). Rare mutations in α1-antitrypsin convert it into a much more powerful contact system inhibitor than C1-INH [32, 33], and mutagenesis studies show that recombinant variants of this mutant α1-antitrypsin can be used to attenuate bradykinin-dependent inflammation in vivo [34].

#### The BK Forming Cascade

The BK-forming cascade, as depicted in Fig. 1, was initially considered solely a fluid-phase, protein-interactive phenomenon. However, the observation that HK demonstrates zinc-dependent binding to the surface of platelets [35] and endothelial cells [36] leads to the concept that all of major constituents needed to produce BK in plasma interact with vascular endothelial cells, via proteins expressed on the cell membrane. These were identified as globulated C1q receptor (gC1qR) [37], cytokeratin 1 [38], and urokinase plasminogen activator receptor (uPAR) [39]. They exist as two bimolecular complexes, namely, gC1qR-cytokeratin 1 and cytokeratin-1-uPAR [40]. gC1qR and uPAR do not interact directly. Those bimolecular complexes bind either HK or FXII, but not both [41]. Although comparative studies of binding employing the complexes have not been done, we have surmised that gC1qR-cytokeratin 1 binds primarily HK [42–44], while cytokeratin-1-uPAR binds primarily FXII [45]. Individually, gC1qR binds HK and FXII, cytokeratin-1 binds primarily HK, and uPAR binds primarily FXII [42, 45], but can also bind cleaved HK (cHK) and to a very limited extend to native HK [39].

**Fig. 2:**
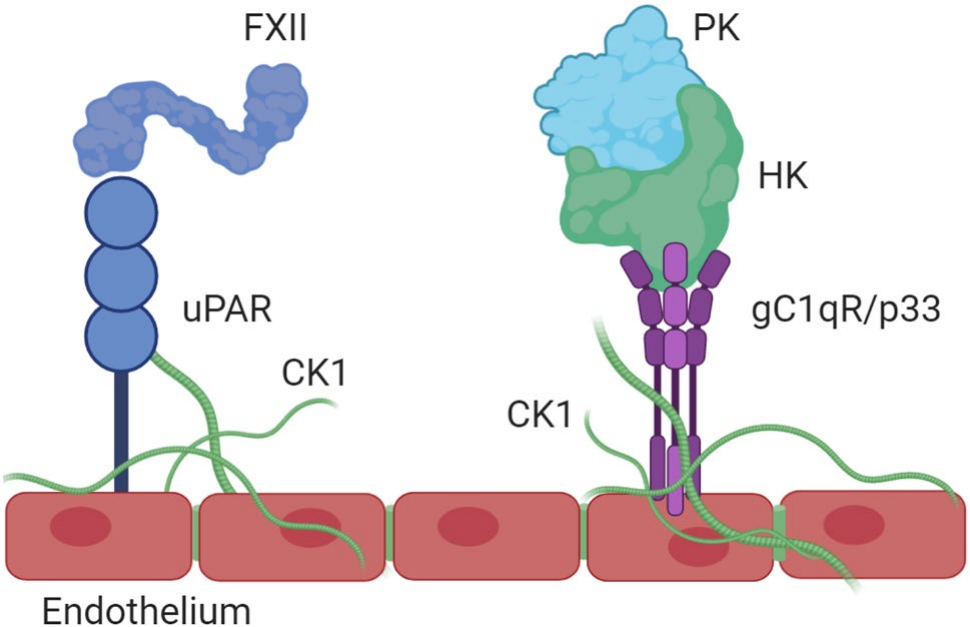
Assembly of the contact system on the surface of endothelial cells. Abbreviations: FXII; factor XII, PK; plasma prekallikrein, HK; high molecular weight kininogen, uPAR; urokinase plasminogen activator receptor, CK1; cytokeratin 1, gC1qR; receptor for globular heads of C1q

Since many of the individual proteins bind both constituents of the complexes, it is possible that some HK and FXII bind to both biomolecular complexes, but with different ratios. Plasma kallikrein is brought to these complexes by virtue of its attachment to HK. A diagram depicting key reactions along the endothelial cell surface is shown in Fig. 2. The crystal structure of gC1qR to which either FXII or domain 5 of HK is bound has been resolved [46]. In this study, it was elucidated that FXII binds to gC1qR through its fibronectin type II domain in a Zn^2+^-dependent manner. As this domain regulates zymogen activation, we propose that gC1qR binding primes FXII for activation [14]. At the same time, each gC1qR trimer contains one binding site for HK (domain 5). As a result, it can assemble both HK and FXII simultaneously [46]. This sets the stage for cell-surface based contact system activation.

Activation of FXII occurs minimally upon binding to endothelial cells [47–49] based on the cell’s “surface” characteristics. Thus, the binding to macromolecules described above is not the same as binding to negatively charged inorganic molecules such as polyphosphate, nucleic acids, and endotoxin [50, 51]. Yet, binding of the PK-HK complex to endothelial cells at normal body temperature of 37 °C leads to production of PKa in the absence of FXII. Here, in a FXII-independent manner, heat shock protein-90 (HSP-90), a non-protease stress protein, is secreted from vascular endothelial cells and interacts with the complex of PK-HK, resulting in PKa production [52]. In contrast to the activated FXII pathway, this optional circuit does not produce PKa unless the PK is bound to HK. Interestingly, a similar observation has been reported for prolyl carboxypeptidase (produced locally by endothelial cells), which activates only the PK-HK complex, but not PK alone [53]. HSP-90 secretion can be stimulated by interaction of endothelial cells with estrogen, interleukin 1, or TNFα [54].

### Inferences Relevant to HAE

These observations raise issues regarding the initiation of an HAE attack. While FXII is always present, could the activation of endothelial cells as described above, function as an initiator of BK formation followed by PKa activation of FXII? or do surface events as we observe for plasma reactions, and serve as the first step? [55]. Certainly, BK interaction with its B2 receptor on endothelial cell is the critical final step to cause an increase in vascular permeability resulting in angioedema [56].

### Plasma Constituents Involved in HAE Pathophysiology

#### Coagulation Factors

Blood coagulation is typically divided into the intrinsic and extrinsic coagulation cascades. These differ in the first few steps with factor X (FX) as a division point [57]. “Extrinsic” means initiation by a biologic substance other than a plasma protein, which in this instance is tissue thromboplastin (TF-tissue factor). This is a cellular product that interacts with factor VII to yield the TF-factor VIIa complex [58], which in turn functions as an activator of FX. The intrinsic coagulation cascade involves contact with a negatively charged macromolecular surfaces, such as vascular endothelial layers in vivo, or glass test tube silicates, kaolin, or celite which all bind FXII in vitro [59]. All the proteins that attach to that surface are considered part of the contact system which in modern terms include FXII, FXI, PK, and HK. Thus, it refers to the same constituents as the BK forming cascade (the KKS), but for the addition of FXI. Once FXIIa forms, there is conversion of FXI to FXIa, then FIX to FIXa (the first calcium-dependent step) and activation of FX by FIXa with thrombin-activated factor VIII as a cofactor.

This review of HAE-C1-INH has activation of FXII as a crucial point. However, FXII cannot be activated normally in the absence of PK or the absence of HK [60]. It has been known since the 1950s that FXII-deficient plasma will not clot using in vitro assays such as in the activated partial thromboplastin time assay (aPTT). Fletcher factor (PK)–deficient plasma was shown to be identical to PK-deficient plasma [61] and has an unusual coagulation aPTT profile. It is markedly abnormal when incubated with the surface material for 2 min prior to re-calcification. But as the time of incubation with the surface increases, prior to re-calcification, the aPTT shortens and approaches normal. Thus, it is plausible that the plasma autocorrects on prolonged incubation with a surface. This appears to be due to the absence of the PKa feedback activation of FXII which quantitatively accounts for most FXII activation when a aPTT is performed [62]. In the absence of PK, further FXII activation [63] can occur only by auto-activation initiated by the trace activity known to be present in zymogen FXII [64]. Some years later, HK-deficient plasma was discovered, and its aPTT was found close to being as abnormal as FXII deficient plasma, but it does not autocorrect. The explanation is multifactorial. First, conversion of PK to PKa is slower in the absence of HK so that the rate of PKa activation of FXII is also diminished. Second, FXI activation on a surface is highly dependent on HK, so clotting does not proceed in HK-deficient plasma [27, 28]. Thus, PKa and HK can be considered clotting factors.

#### Fibrinolysis

Fibrinolysis is also FXII-dependent [62], and the rate of plasmin formation in FXII-deficient plasma parallels what we see when aPTT is performed. It is markedly depressed in plasma deficient in either FXII or HK and it is abnormal, but autocorrects when PK-deficient plasma is tested. While tissue plasminogen activator (tPA) and urokinase (uPA) are potent, well-characterized plasminogen activators in the vascular tissues, no comparable factor is circulating in the human plasma. FXIIa itself [65], PKa [66], and FXIa [67] have all been reported to directly convert plasminogen to plasmin; however, the main pathway may be PKa activation of a small amount of pro-urokinase present in the plasma and the urokinase produced, converts plasminogen to plasmin [68].

#### Complement

A key link between the BK-forming pathway and complement activation that is also relevant to the pathogenesis of HAE-C1-INH is the ability of factor βFXIIa (FXIIf) to activate C1r and to a lesser degree C1s (both comprises the esterase complex of C1) [69, 70] (Fig. 1). The consequence is activation of C4 and then C2. This may be the explanation for the changes in C4 and C2 that occur during attacks of angioedema in such patients, and what makes low C4 a hallmark of C1-INH deficiency. In short, factor βFXIIa continues BK formation, but loses the ability to clot (through FXI activation) and gains the ability to activate the classical complement pathway.

Indeed, the key plasma abnormality characteristics of types I and II HAE are low C4 level in about 95% of patients, abnormal function (activity) of C1-INH, and the instability of patients’ plasma even in the absence of an initiating (activating) surface. This is evidenced in the laboratory when BK in patients’ plasma is gradually produced upon prolonged incubation at 37 °C.

#### The Paradox of FXII Activation Without Pro-thrombotic Tendency in HAE

One paradox noted early on is that although FXII, PK, and HK all interact to optimally generate FXIIa in order to convert FXI to FXIa, a deficiency in any one of these three proteins does not lead to bleeding. Furthermore, a deficiency of FXI, the next protein in the sequence, does cause spontaneous bleeding, particularly when plasma levels are very low, and is therefore considered a form of hemophilia [71]. One thesis to explain this anomaly is that thrombin (Factor IIa) can directly activate FXI and bypass these three proteins [72], but plasma experiments cast doubt on this explanation [73]. While in vitro clotting (the aPTT) is markedly abnormal for FXII and HK deficiencies, the original FXII-deficient patient (Mr. Hageman) died of a pulmonary embolus—therefore he clotted! A defective FXII-dependent fibrinolysis (described above) might be a plausible explanation. In HAE, all these factors are activated; however, in vivo thrombosis is not seen. Nevertheless, there is evidence for activation of the entire coagulation cascade concurrently with increased fibrinolysis as well. Thus, prothrombin fragment 1–2 levels are elevated during HAE attacks [74] implying a conversion to thrombin, the final enzyme of the coagulation cascade, which explains the strikingly elevated D-dimer levels [75, 76]. This reflects thrombin action on fibrinogen to form fibrin and digestion of crossed-linked fibrin clots by plasmin. Further, while such activation is ongoing [75], D-dimer blood levels also appear to reflect disease activity and may explain why provision of C1-INH could dampen HAE disease activity [77].

We offer two possible explanations to this contradicting phenomenon. Firstly, since BK interaction with the kinin B2 receptor on endothelial cells may result in release of TF, thereby producing thrombin via the extrinsic coagulation cascade. This involves factors VII, X, prothrombin, cofactor factor V, and fibrinogen (PMID 27826093). Secondly, endothelial cells can also release tissue plasminogen activator (tPA) and/or uPA to convert plasminogen to plasmin, which could account for the above abnormalities. The location where this coagulation/fibrinolysis cycle takes place is critical. If it takes place in the extravascular space, this is not accompanied by thrombotic events. On the other hand, if it takes place in the intravascular compartment, the rate and concentration of thrombin in the presence of inhibitors such as antithrombin (III) might prevent thrombotic events.

**Fig. 3:**
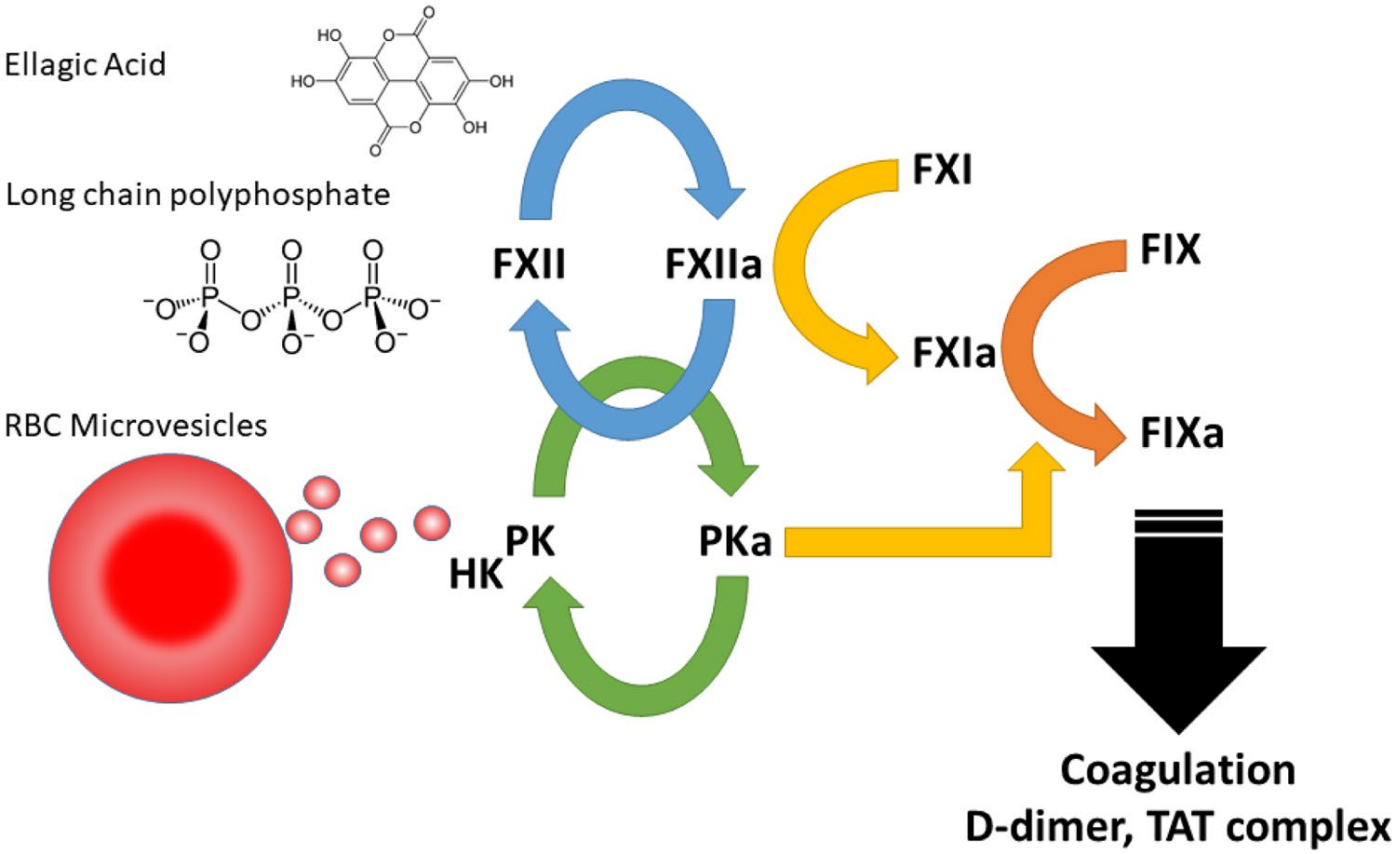
Plasma kallikrein (PKa) as a clotting factor. Abbreviations: FXII factor XII, FXIIa activated FXII, PK plasma prekallikrein, PKa activated plasma kallikrein, HK high molecular-weight kininogen, FXI Factor XI, FXIa activated FXI, FIX Factor IX, FIXa activated FIX. RBC red blood cell, TAT-complex thrombin-antithrombin complex

A second, very interesting recent observation is that PKa can bypass FXI (or act along with it) to activate FIX and allow the intrinsic coagulation pathway to proceed downstream. This hypothesis is strengthened by recent identification of role for PKa as a clotting factor during contact activation by anionic surfaces. It is able to activate Factor IX, independent of FXI. This helps to explain why the bleeding phenotype of FXI deficiency is relatively mild [78]: PKa can substitute for FXIa. In addition, it was recently reported that vesicles from red blood cells, which are generated during ex vivo storage, present a currently unidentified protein-based activator of FXII and PK [79]. Also, in this setting, it was found that PKa can act as a direct FIX activator. These two studies are in good correspondence with earlier studies that implicated a FXI-independent way to activate FIX after contact activation by long-chain polyphosphate polymers, derived from bacteria [80]. These combined studies (schematically represented in Fig. 3) suggest that in addition to its key contribution to BK production, PKa has the ability to act as a “backup” clotting factor. It should be mentioned that FIXa is a particularly remarkable enzyme: after being activated, it has an extremely low activity in solution, making it both undetectable and insensitive to inactivation by antithrombin [81]. This unique feature means it is able to escape inactivation in the circulation, which may be relevant in the chain of events leading to vascular endothelial hyperpremeability. FIXa activity increases over 1.000.000-fold when it meets anionic phospholipid surfaces at sites of injury without a need for an additional activation step to rapidly initiate coagulation. In conditions with excessive PKa activity, such as HAE, it is possible that these combined mechanisms contribute to systemically and sustained elevated levels of coagulation parameters [76, 82].

## Conclusion

Activation of the plasma contact system can lead to the activation of the intrinsic pathway of coagulation and the formation of the inflammatory peptide bradykinin. While the contact system is immaterial for normal hemostasis, in vivo models show a central role for FXII in thrombosis. Inhibition or knocking out FXII can prevent thrombosis in these models, but whether this can be translated to humans is not yet clear. In comparison, the role of the contact system as the producer of bradykinin in hereditary angioedema has been proven in both in vivo models and patients. Nonetheless, coagulation, fibrinolysis, and bradykinin production seems to be intertwined in HAE, where D-Dimer levels correlate with disease activity. In this review we explored the molecular mechanism of contact system assembly and activation on the endothelial surface. Furthermore, we discuss the possibility that bradykinin production may indirectly lead to the activation of the intrinsic pathway of coagulation via activation of the bradykinin receptor of by activation of FIX by PKa. Furthermore, we discuss the possibility that bradykinin production can be facilitated/augmented by secretion of endothelial cell products such as HSP-90 or prolylcarboxypeptidase and that activation of FIX by PKa (bypassing FXI) is yet another contact activation-dependent pathway leading to thrombin formation.

Collectively, these mechanisms may explain the complicated blood clotting pathogenesis of bradykinin driven diseases such as seen in HAE.

## Abbreviations

aPTT: Activated partial thromboplastin time
ANGPT1-HAE: HAE with angiopoietin mutation
BK: Bradykinin
C1-INH: C1 esterase inhibitor
C1-INH-HAE: HAE with C1-INH deficiency
cHK: Cleaved HK
EGF: Epidermal growth factor
FXII: Coagulation Factor XII (Hageman factor)
FXI: Coagulation Factor XI (Rosenthal factor)
FXII-HAE: HAE with Factor XII mutation
HSP-90: Shock protein-90
HAE: Hereditary angioedema
HK: High molecular weight kininogen
KNG1-HAE: HAE with kininogen 1 mutation
LK: Low molecular weight kininogen
MYOF-HAE: HAE with myoferlin mutation
nC1-INH-HAE: HAE with normal C1-INH
PK: Prekallikrein (Fletcher factor)
PKa: Plasma kallikrein
PLG-HAE: HAE with plasminogen mutation
TF: tissue factor (tissue thromboplastin)
tPA: Tissue plasminogen activator
TNFα: Tumor necrosis factor alfa
uPA: Urokinase plasminogen activator
U-HAE: HAE with unknown mutation and unknown cause
